# Development of an ethogram/guide for identifying feline emotions: a new approach to feline interactions and welfare assessment in practice

**DOI:** 10.1186/s13620-021-00189-z

**Published:** 2021-03-25

**Authors:** Sandra Louise Nicholson, Roslyn Áine O’Carroll

**Affiliations:** School of Veterinary Medicine, University College Dublin, Dublin, Ireland

**Keywords:** Feline emotions, Feline behaviour, Feline welfare assessment, Risk (feline handling), Animal behaviour education

## Abstract

**Background:**

An accurate assessment of feline behaviour is essential in reducing the risk of handler injury and evaluating/improving feline welfare within veterinary practices. However, inexperience and/or suboptimal education in feline behaviour may cause many veterinary professionals to be ill equipped for this. In addition, busy veterinary professionals may not have time to thoroughly search the literature to remediate this deficiency. Upon searching the literature, terms such as aggression and stress predominate, but these do not completely represent the rich mental lives that cats are now understood to have. Emotions have recently emerged as an alternative approach to animal behaviour/welfare assessment. However, few resources describe how to identify them, and positive emotions are particularly neglected. In addition, no simple, broad, and concise guide to feline emotions currently exists within the research literature. Therefore, this research aimed to develop a straightforward and clear reference guide to feline emotions (ethogram) to aid veterinary professionals in interpreting feline behaviour in practice and for use in veterinary education.

**Results:**

Five primary emotions were identified and defined for domestic species (fear, anger/rage, joy/play, contentment and interest). A feline emotions guide (feline emotions ethogram) was created. Three hundred and seventy-two images were captured of feline behaviours indicative of emotional states. Of these, ten of the best quality and most representative images were selected to illustrate the guide (two of each emotional state). The feline emotions guide and its associated images were subsequently validated by two feline behaviour experts.

**Conclusions:**

Following slight modifications, the emotions definitions yielded during the feline ethogram design process may be transferable to other domestic species. The feline emotions ethogram/guide itself may be particularly helpful for distinguishing immediate motivations and customising patient care within short- term veterinary contexts. Hence, its use may improve feline welfare and feline handling/interactions. However, the guide will need to be reliability tested/ tested in the field and may require adaptation as the feline emotions’ knowledge base grows. In addition, novices may benefit from exposure to more images of feline emotional state, particularly those involving mixed emotions. Freely available online images and videos may be sourced and used to supplement the accompanying image bank.

## Background

Veterinary professionals in small animal or mixed practice regularly assess feline behaviour in order to safely interact with cats and monitor their wellbeing. Errors in assessment may lead to personal injury and/or negative animal welfare consequences. Cat-attack injuries are very common in veterinary nurses [1, 2] and veterinarians [1, 3], and they may lead to physical damage, emotional distress [1, 4], work absences [1] and secondary infections [3]. Research has suggested that recently qualified veterinary professionals have the greatest risk of injury. Nordgren et al. [4] found that veterinary nurses with fewer than 5 years’ experience in practice and those younger than 25 years of age were more likely to incur bite wounds than their older, more experienced colleagues. Similarly, Epp and Waldner [3] noted that veterinarians with full-time experience of 5 years or less were more likely to report bite injuries. This may reflect the fact that animal behaviour education [5, 6] and feline handling experience [7] can be limited during undergraduate training. Limited experience with cats may also affect the ability of the veterinary professional to assess feline wellbeing. This is problematic as cats often find aspects of the veterinary environment very challenging [8, 9]. These aspects may include restrictive housing, altered daily routines, noise, and exposure to unfamiliar smells or individuals [8, 9]. The distress experienced could prevent cats from engaging in maintenance behaviours such as eating, drinking, sleeping, and elimination [8]. In addition, distress may alter clinical parameters [8]. The net result is that patient care and animal welfare are compromised. To avoid this, veterinary professionals (particularly trainees or recent graduates) need guidelines on how to assess feline behaviour. Typically, animal welfare assessment involves the use of behavioural and hormonal (cortisol) indicators of stress [10]. However, the stress response is not a specific indicator of poor welfare, as it may be stimulated by positive or negative circumstances [11]. Therefore, new indicators are needed for assessing feline welfare and evaluating the risks of handling/interaction. Emotions may be the key. Studies in neuroscience have provided evidence for the existence of emotions in animals [12, 13]. As emotions influence behaviour [14], an understanding of the cat’s emotional state may help veterinary professionals to predict the risk of handling and plan interactions. They can also provide insight into how the animal perceives its environment [14] and thus are useful tools for assessing animal welfare. However, the literature on feline emotions, although growing, is still limited, and most resources focus on negative (unpleasant) emotions or emotional valence. It is important that positive (pleasant) emotions are not neglected, as their consideration can bring a balanced perspective to safety and welfare assessments. The presence of positive emotions may signal good welfare [15, 16] and suggest that the cat will cooperate with staff. Busy veterinary professionals may not have time to comprehensively review the literature on feline emotions and so may value a tool that summarises and interprets it. In consideration of all this, our project aimed to create a balanced, illustrated, and entry level reference guide to feline emotions (feline emotions guide/ethogram) for use by veterinary professionals and in veterinary education.

## Materials and methods

### Identifying and defining feline emotions

In order to create a feline emotions guide/ethogram, it was first necessary to determine which emotions were valid and recognisable in animals and to clearly define these. A literature search was conducted for this purpose. Scholarly textbooks were accessed for foundational material and academic search engines (University College Dublin (UCD) One Search, Wiley Online Library, Google Scholar, JSTOR, PubMed, Science Direct and Ingenta Connect) were used to find relevant journal articles. The existence of secondary emotions in animals (such as jealousy, pride and empathy) was supported by anecdotal evidence only [17]. Therefore, these were not explored further. However, the existence of primary emotions in animals was strongly supported by neuroscientific studies, as detailed by Panksepp [12, 13]. Therefore, Panksepp’s “emotional circuits” (seeking, fear, anger/rage, lust, care, panic, and play) [12, 13] were used as the basis for the emotions. Anxiety was not distinguished from fear [12, 13] and a further search of the literature revealed no specific neuronal circuitry to support its inclusion. In addition, frustration was not listed as a core emotion [12, 13]. Instead, it was determined to be a state leading to anger/rage rather than a distinct emotion [12]. Therefore, it was not selected as a core emotion for the guide. For our purposes, only emotions directly observable in a clinical setting would be useful. As many feline patients are neutered and/or are housed singly, lust [12, 13] was excluded from the emotions guide. Care [12, 13] was excluded because it would only be seen when nursing queens and their kittens are hospitalised together, and this is not a very common occurrence. Finally, panic [12, 13] was excluded because it was thought to be too difficult to distinguish from extreme fear. The remaining systems (seeking, fear, anger/rage and play) [12, 13] were judged to be appropriate for inclusion. Seeking and play [12, 13] were considered to reflect actions rather than emotions and so new identifiers were sought to replace these terms. As seeking indicates a desire to explore and interact with the environment and obtain resources [13], “Interest” was judged to be an appropriate term. An emotion was also required to represent the pleasure associated with the satisfaction of this desire. This was not included in Panksepp’s emotional circuits [12, 13]. However, interconnected “pleasure centres” have been identified in the brain, and stimulation of these centres causes a “liking” response [18]. This inspired the choice of “Contentment” as a primary emotion**.** Play was renamed “Joy” as per Panksepp’s [12, 13] description of that system. Once selected, the primary/core animal emotions were defined with reference to the literature (see Table 1). Both animal behaviour/veterinary behaviour and human psychology literature (particularly resources on positive psychology) were drawn upon to create these definitions. Each definition included the type of emotion (whether it is a positive or negative sensation). This is customary in psychology and also significant for animal welfare. The general triggers/causes for each emotion were included in the definitions, as this is helpful for assessing and modifying the environment. Each definition was completed by the addition of the behavioural strategies/types used to express emotion. Behavioural strategies are more commonly referred to in the literature than animal emotions. Therefore, this approach was necessary in order to make the connection between emotional states and their specific behaviours. The definitions were originally created in 2016. However, they were further refined and improved in 2019–2020. In particular, “Joy” was renamed “Joy/Play” in response to feedback received from an anonymous reviewer. The reviewer had commented that feline object play may involve a predatory drive rather than simply joy. The definition of “Joy/Play” was also expanded to include the behavioural strategies involved in play (play categories), as described in the literature [25, 26].

**Table 1:** Definitions of feline primary emotions

Emotion	Definition
*Fear*	Negative emotional state [19] caused by immediate perceived danger or the threat of danger [20] and manifested as vigilance and attempts to withdraw or escape [21].
*Anger/Rage*	Negative emotional state [22] caused by the frustrated desire to perform actions/achieve goals [12] (including escape or exploration) or by competition for resources [23]. Manifested as aggression or the threat of aggression [22].
*Joy/Play*	A high intensity positive emotional state [24], which may be internally motivated [23]. Manifested as non-functional behaviours involving physical activity (locomotor play), interaction with other individuals (social play), or interaction with objects (object play) [25, 26].
*Contentment*	A positive emotional state caused by the fulfilment of the animal’s needs and desires and an acceptance of their current state [27]. Manifested as resting [28], calm [29], and affiliative behaviour [29].
*Interest*	A positive emotional state [28], caused by the presence of a novel stimulus or stimulus of salience and/or anticipation of engagement [30]. Manifested as attention and orientation to the stimulus [30, 31] and/or seeking behaviours [12, 13].

### Design of the feline emotions guide/ethogram

The definitions created for each core feline emotion acted as a foundation for designing the behaviour guide. The behavioural strategies were used to find individual behaviours and/or postures/body language associated with each emotion. For example, aggressive or threatening behaviours (towards any target but not associated with predation) were sought as indicators of anger/rage and play behaviours were sought as indicators of joy. During an additional literature search, a behaviour directory (ethogram) for wild and domestic cats was discovered [32]. This directory detailed numerous feline behaviours/postures and also grouped them into broad categories [32]. Some of these categories were similar to the behavioural strategies used in our definitions (affiliative, calm, aggressive and agonistic, exploratory) [32]. Fear was presented as a specific category and play was listed under “active behaviours.” [32] Therefore, this ethogram, created by Stanton et al. [32], was a rich source of information for the design of the feline emotions guide. However, details were also obtained from other sources such as Bradshaw et al. [33], Finka et al. [34], Overall [35], and Shaw & Martin [36]. Vocalisations were omitted, as a single type of vocalisation may be used to express more than one type of emotion [35] and there may be individual differences in vocalisations between cats [37]. The first version of the ethogram was completed in 2016. However, it was later updated (in 2019 and 2020) to include items from more recent academic papers [38–40]. It was also reorganised for clarity and conciseness. To make the guide easy to use, it was divided into sections comprised of body language/postures (eyes, ears, tail, body), actions, risk of handler injury, and risk of welfare issue. Anger/rage and fear were categorised as a high risk to welfare because they are unpleasant emotional states that animals seek to avoid [12, 13]. In addition, they may be aroused by a suboptimal environment involving challenges (anger/rage) [23] or perceived danger (fear) [20]. Interest and joy/play were categorised as moderate risks to welfare. In neuroscientific studies, animals actively seek to stimulate the “Interest” system indicating that this is likely to be associated with pleasant sensations [23] and hence positive welfare. In addition, play is often associated with positive welfare states [26]. However, the unsatisfied motivation to explore or play could result in frustration and ultimately anger/rage. In this case, negative welfare would also result from the unmet expectancies or needs. Contentment was categorised as a low risk to welfare, as by definition it is based on having all needs met [27]. As anger/rage may result in aggression [22], this emotion was categorised as a high risk to the handler. In addition, as frustrated fear, interest or joy/play may lead to anger/rage, they were categorised as moderate risks to the handler. However, contented cats do not perceive an immediate threat to their wellbeing. Therefore, contentment was categorised as a low risk to the handler.

### Image capturing, editing and selection

To support and illustrate the emotions guide, multiple photographs were taken of cats displaying behavioural indicators of each emotional state. These were obtained from within the rescue cattery of “Drogheda Animal Rescue” (DAR) and collected in summer 2016. The study received approval and exemption from a full ethical review by the University College Dublin (UCD) Animal Research Ethics Committee (AREC-E-16-10-Nicholson). For ethical reasons, ill cats were not involved, and negative emotional states were only captured if naturally expressed. However, positive emotional states were encouraged through play, petting, and interaction (when appropriate). A Canon EOS 1000D, Panasonic HDC-SD60, Canon PowerShot SD1200 IS, and a Samsung Galaxy S5 were used interchangeably throughout the three-week shooting period. Good natural lighting created optimal conditions for acquiring images. Flash use or low light levels may have artificially affected pupil size and confounded the interpretation of emotion. Where possible, cage bars were not included in the images to allow for a clear view of the subject. Every effort was made to exclude contextual information such as toys, food etc. from images. It was thought that these could distract from the body language of the pictured cat and/or provide independent “clues” to the cat’s emotional state. Once collected, the images were categorised based on the emotion depicted, and edited (as needed) to reduce blurring and improve contrast. Ten of the best quality and most representative images were selected to illustrate the emotions guide (two images for each emotional state). Images representing a single emotion were preferred to those involving mixed emotions, as it was thought that these would be easiest for a novice to interpret/learn.

### Validation

In autumn/winter 2020, the feline emotions guide/ethogram and associated images were sent to two different individuals for (content) validity testing. These individuals were both certified clinical animal behaviourists (CCABs) from the United Kingdom. A number of changes were made to the feline emotions guide/ethogram as a result of their feedback. “Slapping feet against ground” and the tail position of “Upright and bent over the body” (which were previously listed under anger/rage and taken from Stanton et al. [32]) were removed, as they were deemed inappropriate by the validators. In addition, behaviours involved in friendly greeting or hunting/predation were explicitly categorised as such. A caveat was added that pupil size/shape could be affected by arousal or light levels in addition to emotional state. Finally, one validator also advised that the researchers study their outputs carefully to ensure that they were satisfied with the final product. As a result of this, the researchers added items from more recent literature [38–40] to the feline emotions ethogram.

## Results

### Feline emotions guide/ethogram

Please see the completed feline emotions guide/ethogram (Table 2) below.

**Table 2:** Feline emotions ethogram/guide [32–36, 38–40]

EMOTION	BODY LANGUAGE				ACTIONS	RISK OF HANDLER INJURY	RISK OF WELFARE ISSUE
	EYES	EARS	TAIL	BODY			
**Fear**	Wide open eyes [35, 39] with round dilated pupils [35].^a^ Blinking or half blinking [38]. Or eyes tightly shut [39] or avoidance of eye contact [33, 36, 39]. Gaze to left in mild fear states [38].	Flattened [34] to the side [33, 35, 36] or back [32, 35, 36, 38]. Ear pinnae are not visible [35].	Tucked under the body or wrapped around it [32, 36, 39].	Piloerectio n[36, 39]. Tense muscles [39]. Crouching [32, 38, 39]. Lowered head [36, 39] Standing with arched back [35]. Left head turn in mild fear [38].	Vigilance [39]. Startle [39]. Trembling [32, 39]. Freezing [32, 38, 39]. Hiding [32, 38, 39]. Fleeing/avoidance [32, 38, 39]. Grooming [32]. No maintenance behaviours (eating, drinking, elimination)/sleep [39].	MODERATE	HIGH
**Anger/Rage**	Pupils oblong and dilated [35].^a^ Direct stare [32, 35, 36].	Swivelled sideways [33–35]. Inner pinnae are visible [35].	Lowered and rigid [35]. Held in inverted L shape [36]. Slapping against ground [32]. Rapidly moved from side to side (or up and down) [32] (Tail lash [33]).	Piloerection [32, 36] along spine and tail [36]. Leaning forward [36]. Elevated rump [36]. Standing with arched back [35].	Exposing teeth [32]. Launching at/chasing individual [32]. Attacking with paws or mouth [32]. Displace others [32].	HIGH	HIGH
**Joy/Play**	Pupils dilated/round due to arousal [35].^a^ Or relaxed/soft [32].	Upright and forward facing [36].	Vertical [33]. May take an inverted U shape [35].	“Play face” in kittens: a half- open mouth [32, 40]. Arching spine [32, 40]. Body posture varies.	Locomotor play [40] Climbing [40]. Running. Social play [40] Approaching cat [40]. Jumping [40]. Patting, pawing playmate [40]. Grabbing playmate with forelimbs [40]. Biting playmate [40]. Rolling/presenting belly [32, 40]. Wrestling playmate [40]. Kicking/raking playmate [40]. Chasing playmate [40]. Side stepping or running away from playmate [40]. Object play [40] Rearing to reach object [40]. Pawing [25, 40], batting object [40]. Holding object with paws [40]. Sniffing, licking object [40]. Biting [25, 40], chewing object [40]. Throwing object [40]. Wrestling with object [40]. Predatory: Stalking, chasing, jumping, pouncing on object [40].	MODERATE	MODERATE
**Contentment**	Pupils are small miotic vertical ovals [35].^a^ Half open [36].	Upright and forward facing [32, 36].	Tail relaxed and still [36]. May be erect and slightly curled [32, 35].	Sitting [32]. Lying curled up in circular formation [32].	Stretching [32]. Yawning [32]. Grooming self or other (allogrooming) [32]. Kneading/treading paws [32]. Friendly greeting (nose touching/sniffing, head butting, rubbing face and body against object/individual-allorubbing) [32]. Rolling onto back or from side to side [32]. Nuzzling [32]. Eating [32]. Clawing object [32].	LOW	LOW
**Interest**	Dilated/round pupils [35].^a^ Gaze to right [38]. Observing an individual or object [32].	Upright and directed forward towards stimulus [32, 36]. Ear flick [36].	Depends on context. Horizontal [33]. Tail up/vertical in friendly greeting [33].	Standing on hindlimbs [32]. Resting forepaws against object [32]. Stretching head out forward [36]. Head turn to right [38].	Exploring the area or objects [32]. [32]Sniffing . Lickin g[32]. Pawing [32]. Friendly greeting (touching noses with another cat or rubbing face & body against object/individual-allorubbing) [32]. Hunting (stalking, chasing, pouncing, grabbing, biting) [32].	MODERATE	MODERATE

^a^Please note that pupil size and shape may also be influenced by arousal and ambient light levels

### Images obtained

**Fig. 1:**
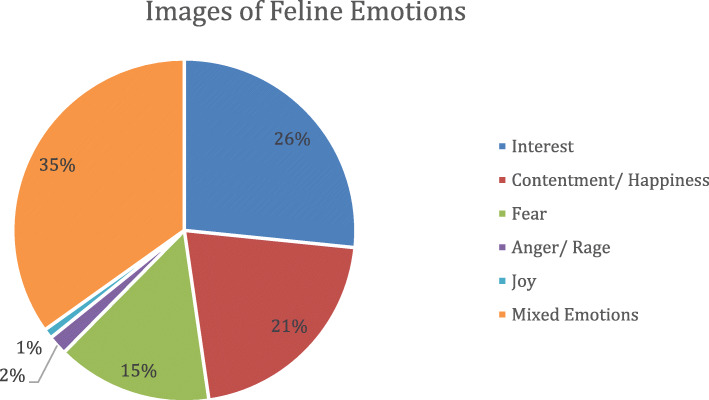
Representation (%) of each emotion in the pool of 109 images

**Fig. 2:**
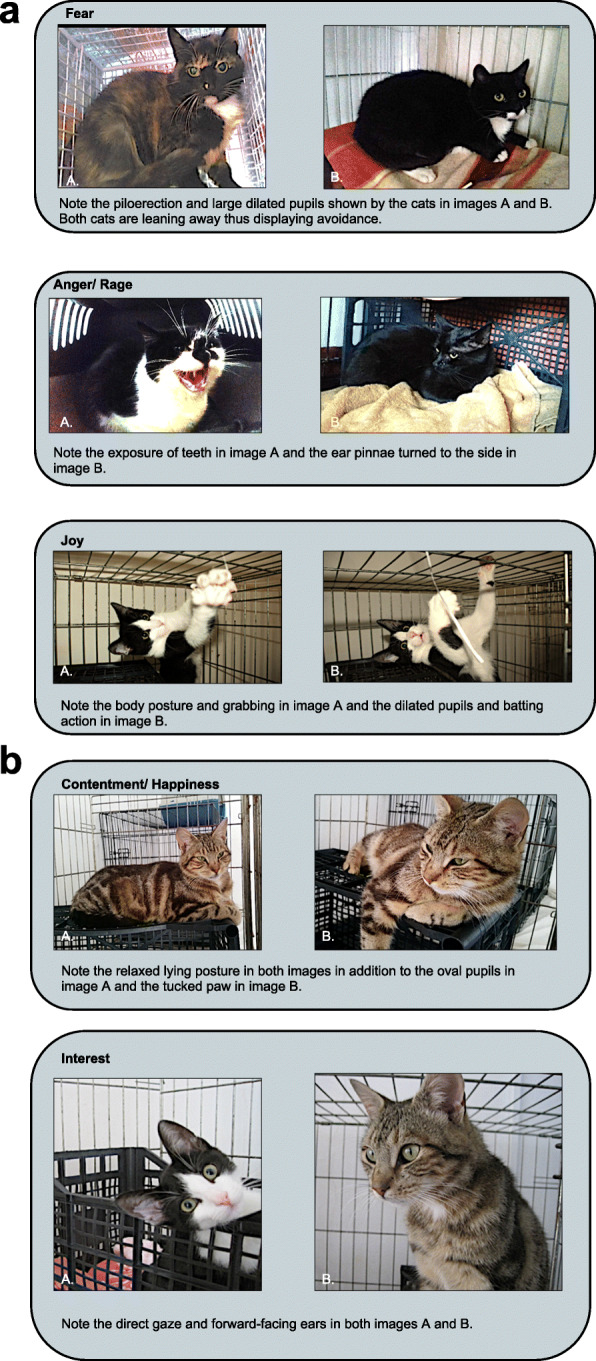
**a** and **b**: Feline Emotions Images

In total 372 images were obtained, and 109 of these were worthy of further consideration. Amongst the 109 selected images, interest (26%), contentment (21%), and fear (15%) were captured most frequently. Anger/rage (2%) and joy (1%) were captured least frequently. Mixed emotions (i.e. involving more than one emotion) were also regularly observed (35%) (see Fig. 1). Ten of the best quality and most representative images were selected to illustrate the emotions guide (two images for each emotional state) Fig. 2.

## Discussion

### Considerations for the use of the research outputs

This research yielded definitions of emotions, and an accessible “entry level” resource for veterinary professionals to use in identifying feline emotions. The definitions and approach used could also be transferable to other domestic species. However, “Lust” and “Care” [12, 13] may first need to be added for species where reproduction is more important (horses or farm animals, for example). Use of the feline emotions guide/ethogram may improve feline interactions, reduce the risk of handler injury, and enhance the welfare of feline patients. It may also be particularly useful for teaching undergraduate students and supporting recently qualified individuals (who are more vulnerable to injury [3, 4]). It amalgamates a multitude of information that is normally scattered throughout the literature and includes details on positive emotions that can often be difficult to find. Unlike other resources, it is designed to be used “live” at the time of interaction to guide feline handling and to respond to any changes in the patient’s wellbeing during procedures. In this way, it can aid the individualisation of care and complement pre-emptive measures, such as nursing care plans and the feline friendly handling and environmental guidelines issued by the International Society of Feline Medicine (ISFM) and the American Association of Feline Practitioners (AAFP) [8, 41]. However, it is intended for use in the short term and is not suitable for determining long-term welfare (cognitive bias or quality of life tools could be used for this instead). To aid novices, the feline emotions guide is organised by the body part/body language indicators or “actions” involved in each emotion. However, the indicators and actions should be used holistically. Some occur with more than one emotional state (piloerection or pupil dilation for example), while others may be more specific (for example play indicators for joy, or flattened ears for fear). The feline emotions guide should be used to distinguish emotions and their associated motivation(s) to determine whether corrective action is necessary. Although both anger/rage and fear signal potential animal welfare issues and/or risk to staff, their underlying motivations differ. Anger/rage is often motivated by frustration (e.g. of exploration, interaction, or accessing resources) [42]. Ideally, frustration would be noted and acted upon prior to anger developing. Frustration manifests as restless behaviours such as pacing, biting or leaning against barriers, scratching, excessive rubbing or pawing, and the disruption of cage contents [39]. With signs of either frustration or anger, cats should be given access to appropriate resources (such as food, water, and litter-trays) and the opportunity to engage in their desired behaviour (such as play, exploration, appropriate stroking) if possible. Alternatively, these cats may simply need a break from handling or disturbance. Unlike anger/rage, fear is motivated by the presence of a perceived danger [20]. Therefore, fearful cats should be given the opportunity to withdraw or hide [43]. Cat cages or carriers could be covered with a towel or blanket, or a hide could be added to hospital cages for this purpose [44]. Distinguishing positive emotions also enables veterinary professionals to better cater to their patients’ needs. An interested cat may be seeking stimulation/interaction and a joyful cat may wish to continue playing, while a contented cat has all its current desires and needs met. The interested cat could be provided with toys, feeding enrichment, appropriate petting, or visual stimulation to prevent frustration. Appropriate play could be continued with the joyful cat. Contentment may be sustained and further promoted by treating the cat as an individual and liaising with its owner to discover its usual routines and preferences. When evaluating feline emotional state, veterinary professionals should be aware that multiple emotions (mixed emotions) can co-exist in the individual at the same point in time. Indeed, mixed emotions were commonly observed during the project fieldwork. However, mixed emotions may also be more challenging to interpret and require more practice to identify. In all cases, veterinary professionals must remain attentive to the cat’s body language to confirm the success or ongoing effectiveness of their interventions.

### Future modifications to the feline emotions ethogram/guide

The feline emotions guide/ethogram has not yet been reliability tested or trialled in the field and some modifications may be required once these processes have been completed. In addition, much still remains unknown about feline emotions, and as the knowledge base grows it may be necessary to adapt the guide. For example, wider use of the Cat Facial Action Coding System (CatFACS), a programme that codes the movements of facial muscle groups [38], could yield new information on the facial expressions involved in feline emotional states. In addition, research in the underexplored area of play in adult cats [40] could find that its characteristics differ significantly from the characteristics of play in kittens. Additional core emotions may be discovered or confirmed in future and require inclusion in the guide. In particular, anxiety was omitted from the feline emotions guide, but it may be necessary to review this decision in future. It was excluded because no specific neuronal circuitry was found to provide evidence for it being distinct from fear. Indeed, the terms “Anxiety” and “Fear” are often used interchangeably in veterinary behaviour. Panksepp and Watt hypothesised that emotional systems may actually consist of “families” of interrelated and gradated emotions rather than isolated core emotions [23]. If so, anxiety may be part of the fear “family” or a form of fear itself. Indeed, both anxiety and fear are responses to perceived danger, and both arise from activation of the amygdala [20]. Anxiety is considered to involve uncertainty about potential danger, while fear is considered to involve apprehension about immediate danger [20]. However, alternatively, some findings from psychiatric research suggest that anxiety is a valid and distinct animal emotion. Animals (usually rodents) placed into experimental contexts involving uncertainty about danger have been found to use behavioural strategies similar to those employed by humans experiencing anxiety [45]. These behavioural strategies included avoidance, escape, vocalisation, hypervigilance, investigation (by sniffing), grooming, and conflicted approach-withdrawal [45]. Interestingly, the performance of some of these behaviours was also reduced following the administration of anxiolytics (benzodiazepines for example) [45]. Although these findings are suggestive, further research is needed to identify anxiety-specific neuronal structures in animals and specific behaviours in cats, prior to anxiety’s inclusion in the feline emotions ethogram/guide.

### Collecting additional images to support the feline emotions ethogram/guide

In future, in addition to making necessary adjustments to the feline emotions ethogram, more images will be needed to complement it. Although many images were collected during the original project fieldwork, fewer than 30% were suitable and of sufficient quality and clarity to be associated with the feline emotions ethogram/guide. Mixed emotions often occurred but these were excluded to simplify the ethogram/guide for beginners. However, these images could be useful for developing further competence in the interpretation of feline emotional signalling. The images selected for use did not illustrate all of the behavioural signs/body language of each feline emotion. In addition, anger/rage and fear were poorly represented, as these emotional states were rarely shown by the cats involved in the study. This may be attributed to the excellent care provided by the shelter staff and/or the considered approach taken by the field researcher. Joy was also displayed infrequently, perhaps because confinement in the shelter cages somewhat impeded play behaviours, or because few young cats/kittens were housed in the shelter at the time. Obtaining images in the field is a rather challenging and time intensive task. However, freely available online images and videos from sources such as Google Images and YouTube respectively could be gathered more efficiently and may also be suitable for use. One of the authors is currently using images from these sources to support the teaching of feline behaviour and her experiences will be reported elsewhere.

## Conclusions

The aim of this project was to create a feline emotions ethogram/guide for use by veterinary professionals and in veterinary education. However, the design process itself also yielded definitions of primary emotions that may be transferable to other species. The completed feline emotions ethogram/guide will aid veterinary professionals in identifying and distinguishing feline emotional states (both positive and negative) in the moment. This may allow motivations to be determined and improvements to be made to both animal welfare and handler health and safety. The ethogram should be used holistically and continuously, and with the understanding that mixed emotions often occur. The feline emotions ethogram will need to be reliability tested and trialled in the field to confirm its usefulness. It may also need to be modified or added to in the future as the research base advances. Finally, additional images should be gathered to enhance its illustration. Images and videos featuring feline behaviour are now freely available online and may reduce the need to capture additional images in the field.

## Data Availability

Data sharing is not applicable to this article as no datasets were generated or analysed during the current study.

## Abbreviations

UCD: University College Dublin
DAR: Drogheda Animal Rescue
CCAB: Certified Clinical Animal Behaviourist
ISFM: International Society of Feline Medicine
AAFP: American Association of Feline Practitioners
CatFACS: Cat Facial Action Coding System
